# Dynamic changes of volatile compounds and bacterial diversity during fourth to seventh rounds of Chinese soy sauce aroma liquor

**DOI:** 10.1002/fsn3.2291

**Published:** 2021-05-12

**Authors:** Lingchang Wang, Kai Zhong, Aimin Luo, Jian Chen, Yi Shen, Xi Wang, Qiang He, Hong Gao

**Affiliations:** ^1^ College of Biomass Science and Engineering and Healthy Food Evaluation Research Center Sichuan University Chengdu China; ^2^ Key Laboratory of Food Science and Technology of Ministry of Education of Sichuan Province Sichuan University Chengdu China; ^3^ Beijing Advanced Innovation Center for Food Nutrition and Human Health Beijing Technology and Business University Beijing China; ^4^ Sichuan Langjiu Group Co., Ltd Luzhou China

**Keywords:** bacterial diversity, different rounds, *Lactobacillaceae*, soy sauce aroma liquor, volatile compounds

## Abstract

Chinese soy sauce aroma liquor (CSSL) is a famous Baijiu. Multiple rounds of fermentation, the characteristic of CSSL processing, contributes to the differences in the quality of the liquor of different rounds. In this study, the grains on cooled, stacked, and fermented stages of 4th to 7th rounds were taken, of which the environmental factors, bacterial diversity, and volatile compounds were comprehensively analyzed. *Lactobacillaceae, Bacillaceae, Thermoactinomycetaceae*, and *Enterobacteriaceae* were the top four families, of which *Lactobacillaceae* dominated the fermented stage of each round. Principal component analysis (PCA) and principal coordinate analysis (PCoA) supported the popular view that the liquors of 3rd to 5th rounds possess the best quality. *Lactobacillaceae* is an extremely critical bacterium for CSSL fermentation. This study provides comprehensive understanding regarding the dynamic changes in fermented grains during the 4th to 7th rounds, which could help to improve the processing technology of CSSL.

## Graphical abstract

By means of metagenomics and metabonomics, the liquor quality degradation from 4th to 7th rounds of CSSL was supported and explained on the perspective of bacterial metabolism. The correlations of bacteria with volatile compounds besides the environmental factors in *Zaopei* were also discussed.

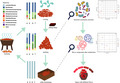

## 1 INTRODUCTION

Baijiu, a transparent strong spirit with 1,000 years of history (Xu et al., 2010), is famous for its distinctive flavor and unique wine culture. The fermentation process of Baijiu differs from other spirits considerably. Baijiu is fermented using multiple microorganisms under solid‐state condition, while west spirits are usually fermented using a single microbe in liquid state (Chen et al., 2014; Jin et al., 2017). With annual consumption of more than 10 billion liters, the Baijiu industry has enormous economic potential.

**FIGURE 1:**
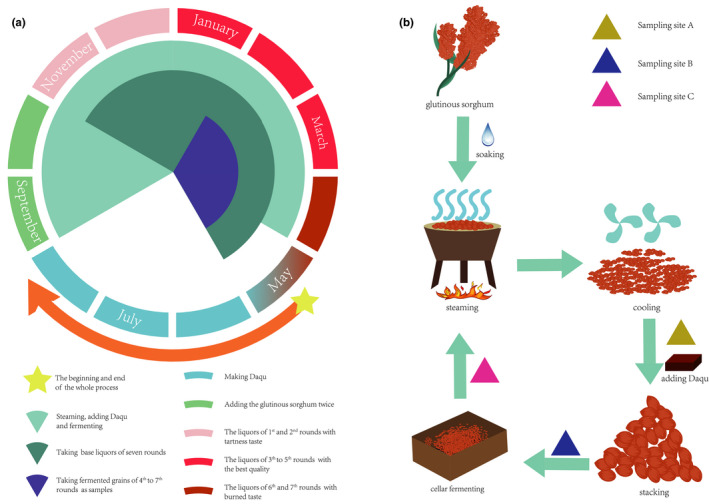
The process technology of sauce aroma liquor. (a) The yearly process of CSSL. (b) A single round of cyclic fermentation and sampling sites

The Chinese soy sauce aroma liquor (CSSL), which undergoes the maximum fermentation, is a typical Baijiu. The yearly cyclic manufacture of CSSL is showed in Figure 1. The entire processing cycle of CSSL requires 1 year for completion, which can be summarized to “adding materials 2 times, steaming 9 times, fermenting 8 times, taking liquor 7 times.” The process involves eight fermentations and seven distillations of the base liquors barring the first fermentation. Empirically, the base liquors in the 1st and 2nd rounds are believed to possess some tart taste, while the liquors in the 6th and 7th rounds have burned or roast taste, called “Xiao Hui” and “Zhui Zao,” while the liquors in the 3rd to 5th rounds, called “Da Hui,” possess the best quality (Yang et al., 2004).

Ester, especially ethyl acetate, was considered to contribute to Baijiu flavor. In addition, various alcohols, aldehydes, acetals, alkylpyrazines, furan derivatives, lactones, and sulfur‐containing, and phenolic compounds were found to be important (Fan and Qian, 2006). In total, 186 flavor substances, including ethyl caproate and caproic acid, were detected in CSSL (Fan, Xu and Qian, 2012). Thirteen compounds with odor activity values (OAVs) greater than 1 were shown to be the key aroma‐contributing substances in Daohuaxiang liquor (Wang et al., 2015). Twenty‐seven key aroma compounds, mainly possessing fruity and floral notes, were successfully used to simulate the aromas of Chinese light aroma type liquors (Niu et al., 2017). However, the main flavor compounds in CSSL remained unclear.

Flavor formation and the corresponding microbial fermentation are the key points for Baijiu to meet the requirements of the modern society (Jin et al., 2017). Brick‐shaped *Daqu*, which is made using wheat, barley, and pea, collects microorganisms during processing, thereafter supplying various microbes and enzymes for fermentation (Du et al., 2019; Xu et al., 2010). The microbes in *Daqu* collected randomly from the environment complicate the process (Wang et al., 2016). The predominant bacteria of light flavored *Daqu* changed from *Pantoea* to *Lactobacillus* after 2 months of aging. Aging rebalanced the interactions of microbes in *Daqu* and was important for improving the quality and ensuring the stability of *Daqu* (Fan et al., 2019). Thirty‐five bacterial families were detected in the three CSSL *Daqu* samples. In particular, *Thermoactinomycetaceae* and *Bacillaceae* were demonstrated to be the common dominant bacteria in three *Daqu* samples (Wang, Ban, et al., 2017; Wang, Du, et al., 2017). In the final stages of Fen *Daqu* fermentation, *Bacillus* and thermophilic fungi became the dominant groups, possibly owing to their tolerance to low water activity and high temperature. Acidity, moisture content, and temperature were reported to correlate with the composition of the microbial communities at different stages (Zheng et al., 2014). Pit muds, the base filler of fermentation pool, affected cellar fermentation by the inner microbes. Related studies have mainly focused on the bacterial and fungal diversity of aging pit muds, which were considered to be better than new ones (Zheng et al., 2013, 2015). *Bacillus* was the predominant class in the early stage of strong aroma style fermentation; subsequently, *Lactobacillus* became predominant when microorganism diversity reduced with fermentation (Wang et al., 2008). However, studies regarding the different rounds of CSSL are scarce because of their complexity.

The bacteria of Maotai liquor cellar fermentation are primarily obtained from four main sources: *Daqu*, air, pit muds, and sorghum (Wang et al., 2016). The microorganisms of *Daqu* or pit muds do not present the complete profile of cellar fermentation (Wang, Ban, et al., 2017; Wang, Du, et al., 2017). Studies on fermented grains (*Zaopei*), which act as the bridge between fermentation microbes and liquor, are lacking. Furthermore, previous studies have always separately analyzed the microorganisms or volatile compounds. The dynamic changes in liquor quality should be investigated for further development of the CSSL industry. In order to verify empirical points and explore the potential relationship among various biochemical indicators, the fermented grains of 4th to 7th rounds of CSSL were chosen and, the environmental factors, volatile compounds, and bacterial diversity of which were comprehensively analyzed.

## 2 MATERIALS AND METHODS

### 2.1 The whole process cycle and sampling rounds

The CSSL process cycle lasted 1 year (Figure 1a). We used the Chinese lunar calendar to match the customs of the people working in this system. *Daqu,* a brick with abundant microorganisms, is made at the beginning for fermentation. The *Daqu* was completed in September, when the winemakers began to add the grains. The grains were divided into two equal parts, and the second time adding was 1 month later after the first time. Cyclic solid fermentation is the characteristic of CSSL. The microorganisms and chemical compounds in fermented grains are changed in stepwise manner. The samples from the 4th to 7th rounds were collected to investigate the microorganisms, volatile compounds, and environmental factors.

### 2.2 Materials and sampling sites

Samples were obtained from CSSL brewing workshop at Erlang Town (Gulin city, Sichuan province, China). The fermented grains were selected as the materials as they are crucial both for the microbes and volatile compounds. Three sites were sampled in each round (Figure 1b). A, B, and C indicated cooled, stacked, and cellar‐fermented, respectively. Stacking or stacking fermentation continued for about 3 days. The microbes from *Daqu* and the environment proliferated and were mixed during stacking. Cellar fermentation in the fermentation pool continued for approximately 30 days. Each sample for sequencing was a mixture of fermented grains from three sites selected randomly, while other biochemical parameters were measured using at least three parallel samples.

### 2.3 Biochemical methods

Acidity, esterification, and starch, amino nitrogen, and moisture content were quantified according to QB/T4257‐2011 (General Methods of Analysis for *Daqu* of China). Proteinase activity was quantified according to SB/T10317‐199 (Measurement of Proteinase Activity of China) with Folin's reagent and the pH was adjusted to 3.0 using lactic acid‐lactate buffer solution. Residual sugar was estimated as follows: 10 g grains were soaked in 100 ml pure water for 30 min; the solution was stirred every 15 min and filtered using a 0.22‐μm filter membrane. The sugar content was measured according to GB/T 15038‐2006 (Analytical Methods of Wine and Fruit Wine of China) using the soluble sugar method.

### 2.4 DNA extraction and PCR amplification

DNA was extracted using the soil DNA isolation kit (FOREGENE, Chengdu, China) according to the manufacturer's instructions. The V3–V4 regions were amplified using bacterial primers 338F (ACTCCTACGGGAGGCAGCA) and 806R (GGACTACHVGGGTWTCTAAT) (Li et al., 2017). The PCR system contained 0.5 μl DNA template, 1 μl 2.5 mM dNTP, 2.5 μl 10× buffer, 0.5 μl 10 μM of each primer, and 1 U Taq DNA polymerase. The PCR started with pre‐denaturation at 95°C for 5 min, followed by 25 cycles of denaturation at 95°C for 30 s, annealing at 50°C for 30 s, and extension at 72°C for 40 s, with final extension at 72°C for 7 min. The PCR products were separated using agarose gel electrophoresis (1%) with 4 μL sample loading at 150 V for 15 min and then purified and recycled using the SanPrep column DNA gel extraction kit (Sangon Biotech, Shanghai, China).

### 2.5 Illumina sequencing and bioinformatics analysis

The purified PCR products were sequenced on Illumina Hiseq2500 at Biomaker Company (Beijing, China). The raw sequencing data have been submitted to NCBI SRA database with submission number SUB8652827. The raw sequencing data were saved in the Fastq format. Next, FLASH (version 1.2.7) and QIIME (version 1.9.1) were used to merge the pair‐end data, and the low‐quality sequence and chimeras were filtered. Clean data were classified into operational taxonomic units (OTUs) beyond at least 97% similarity. The OTU table was generated for subsequent analysis.

### 2.6 Volatile compounds extraction and analysis

#### 2.6.1 Initial extraction and Liquid–liquid extraction (LLE)

A 57% ethanol aqueous solution (v/v) was used as the extractant. Sample (5 g) and 50 ml extractant were mixed by turn in the triangular bottle, soaked, and shaken well. After ultrasound extraction at 25°C, the sample was centrifuged at 10,000 r/min for 10 min. The supernatant was filtered using a 0.22 μm membrane for LLE.

Liquid samples (25 ml of each) were diluted to 10% ethanol (100 ml) which adjusted by boiled, deionized water. The diluted liquor samples were saturated with sodium chloride. Next the solution was extracted three times with dichloromethane. The subsequent extracts were dried by adding 10 g anhydrous Na_2_SO_4_ overnight. Finally, extracts were slowly concentrated to 200 μl under a gentle stream of nitrogen for GC‐MS analysis.

#### 2.6.2 Gas chromatography–mass spectrometry (GC‐MS) analysis

Volatile compounds were separated and identified on GCMS‐QP2010 SE (Shimadzu, Japan) according to previous reports with a minor modification (Xiao et al., 2014). Chromatographic separations were performed on a ZB‐Waxplux column (60 m × 0.25 mm × 0.25 μm). Each fraction (1.0 μL) of the concentrated sample was analyzed by GC–MS. The temperature of the GC injector was set at 250°C. The carrier gas, helium (99.999%), was circulated at 1 ml/min in the constant flow mode with 20:1 split ratio. The column temperature program for GC column was as follows: the initial temperature of 40°C (held for 1 min), increased to 150°C by 4.0°C/min (held for 10 min), increased to 210°C by 10°C/min(held for 18 min), and increased to 230°C by 20°C/min(held for 5 min). The ion energy for electron impact (EI) was 70 eV. Mass spectroscopy was performed at 230°C ion source temperature with a mass scanning range of 35–500 m/z.

The constituents were tentatively identified by matching mass spectrum with NIST05 spectrum database and verified by comparison of their retention indices (RI) with the RI of standards or reported in literatures. The quantity of each component was figured using the internal standard method with n‐amyl acetate, 2‐ethyl butyric acid, and tert‐amyl alcohol or external standard samples. All standard chemicals were analytical reagent and at least 97.0% purity.

### 2.7 Statistical analysis

The biochemistry data were arranged and analyzed using Excel 2013. Variance analysis was conducted using SPSS (19.0). OTU table and purified data were analyzed using R (3.51). Visualization was conducted using “vegan,” “pheatmap,” and “ggplot2” of R. Network diagram was drawn using Cytoscape (3.6.1).

## 3 RESULTS AND DISCUSSION

### 3.1 Volatile compound profiles of different fermented grains

The volatile compounds were detected using GC‐MS. In total, 70 volatile compounds were identified and clustered into six different groups according to their chemical structures, namely 22 esters, 18 alcohols, 14 acids, 8 aldehydes, 4 ketones, 2 phenols, and 2 pyrazines (Table S1). Esters are important and abundant flavor compounds in CSSL, followed by alcohols and acids. This result was consistent with those of previous studies (Fan et al., 2011).

Generally, the concentration of esters, alcohols, and acids increase after cellar fermentation, which account for most of the volatile compounds. Many small molecules were generated during 1‐month cellar fermentation. Ethyl acetate is an important compound contributing to flavor with fruity smell (Zhu et al., 2020), the levels of which was high in 4A and 4B samples, while it was low in other samples. This might imply that the living environment had changed and was not suitable for some ethyl acetate‐producing or ethyl acetate‐utilizing microbes. Ethyl phenylacetate, which has honey aroma (Zhu et al., 2020), presented a similar trend with ethyl acetate. In terms of fermentation rounds, ethyl lactate concentration was low before cellar fermentation but increased sharply subsequently. High level of methanol was produced after 5 rounds of cellar fermentation. Methanol, a colorless volatile compound with mild alcohol odor, is toxic to humans and is readily absorbed by ingestion. But it will continuously decreased over the course of storage (Zhu et al., 2016). Ethyl butyrate level was always low, which was consistent with the results of a previous report (Yang et al., 2004). These results indicated that the quality of the liquors deteriorated with rounds added.

### 3.2 Depth and quality of high‐throughput sequencing

Currently, high‐throughput sequencing is used for investigations regarding food microbiology, and the quality besides depth of sequencing affects the subsequent statistical analysis. The results of high‐throughput sequencing in this study were reliable, as the Q30 of all the samples, indicating the percentage of data with mistake probability <0.001, exceeded 90%. Generally, Q30 should be at least more than 85%. The reliability of data was presented as rarefaction curves of observed OTUs and the Shannon index. The quality of the sequencing data is listed in Table S1. The PCR and sequencing steps generated differences and randomness; hence, the clean data of the 6A group was considerably lesser than the average, which does not indicate that the actual number of bacteria was low (Martin and Linacre, 2020). Shannon index is a parameter that reflects the diversity of a single sample. The rarefaction curves are shown in Figure S1. The relationship between sampling numbers and observed OTUs or Shannon indexes reflects the depth of sequencing. High slope of the curve indicated that the OTUs increased with sequencing depth. In contrast, a smooth curve indicated that the OTU number and diversity will not change significantly even if the sequencing depth is increased, which might represent the majority of the sample. All curves were smooth when the sequencing number exceeded 20,000 (Figure S1). The clean data of most samples were beyond the sequencing number of 25,000 (Table S2), indicating that the sequencing results can reflect most characteristics of our samples.

### 3.3 Alpha diversity and structure of bacteria

The structures of the grains fermented by bacteria were expressed using stacked bar plots at different levels. The alpha diversity of 12 samples is shown in Table S3. Results showed that all C samples had lower alpha diversity. The alpha diversity of three B samples first increased and then decreased in the 5th round sample. This can be explained as follows: the OTUs of the 5B sample decreased slightly; although the OTUs had declined after the 5th stacking, the Simpson index and Shannon index increased. The Shannon and Simpson indexes were affected by the evenness of community, indicating that the structure of the community became more symmetrical, although some very low OTUs had disappeared. The alpha diversity of each round presented an increasing trend before cellar fermentation, which was in agreement with the results of a previous study (Cheng et al., 2014). Stacking is also called “the second making of *Daqu,*” as the microorganisms in *Daqu* spread out in the brick and rebuild the structure of community during this period. With the mixture of *Daqu* and stacking, the alpha diversity index enhanced and microorganisms increased for cellar fermentation. All C samples had low alpha diversity, which was because the environmental factors affected the microbes with progress in fermentation, some of which occupied most parts of fermented grains (Zheng et al., 2014). This conversion produced thousands of metabolites and left fermented grains with homogeneous bacterial diversity. Adding *Daqu* and stacking in the next round made the bacterial diversity revived and prepared the system for the next cellar fermentation.

**FIGURE 2:**
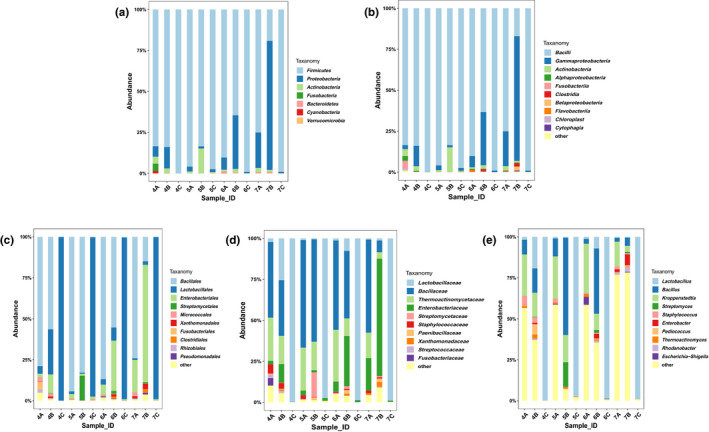
The bacterial diversity of 12 samples at different levels. (a)At phylum level. (b) At class level. (c) At order level. (d) At family level. (e) At genus level

The structure of bacterial community was shown in Figure 2. Ten of the most dominant strains were represented by the numbers of OTUs except Phylum, which had only 7 categories. Generally, at all levels, the diversity of four cycles presented a trend of increase during stacking and decrease during fermentation. At the phylum level (Figure 2a), in terms of cooled stage, the samples of 6th and 7th had quite a small part of microbes belonged to *Proteobacteria*, which had an obvious enhancement after stacking. It might be a reason to explain the difference of quality of these rounds. As the majority of them had the same order, the same trend was observed at the class level (Figure 2b). At the class level, *Bacillus* was divided mainly into *Bacillales* and *Lactobacillales*. As shown in Figure 2c, *Bacillales* mainly existed before fermentation and *Lactobacillales* existed after fermentation. At the family level, all samples showed the best classification (Figure 2d), while the classification included many undefined types at the genus level (Figure 2e). Thus, the taxonomy at the family level was selected to calculate the relationship with small metabolites, which were mainly generated during processing. At the family level, *Bacillaceae* and *Thermoactinomycetaceae* were abundant in 4A and 4B samples. *Lactobacillaceae* was the most advantageous bacteria in all C samples, and it was reported to correlate highly with the high level of ethyl lactate (Gobbetti and Minervini, 2014). The overall trend of changes suggested that the bacterial structure of the fermented grains dramatically change during the fermentation process, which would explain why CSSL requires multiple rounds fermentation.

### 3.4 Volatile compounds and bacterial diversity

Dimension reduction analysis was used to describe the differences between all samples. Principle component analysis (PCA) can reduce the dimensions of multidimensional data to easily acquire the most information from the raw data. Principal component ordinate analysis (PCoA) is similar to PCA; however, the value of the distance is used to plot the graph. Herein, the PCoA was plotted using the Bray–Curtis distance.

**FIGURE 3:**
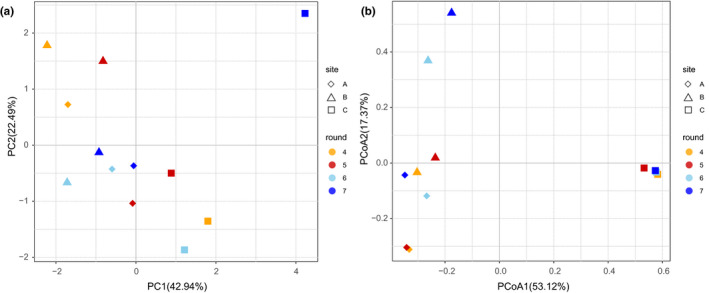
The differences of volatile compounds and bacterial diversity among four rounds. (a)The PCA scatter plot of volatile compounds. (b)The PCoA scatter plot of bacterial diversity based on Bray–Cutis distance

As depicted in Figure 3a, there was an obvious cluster on the left, which indicated that these samples had similar volatile component structures. As the fermented grains on fermented stages were used to distill liquors, the C samples were the main objects. All C samples were on the right side of the cluster that contained the A and B samples, indicating that the volatile components had apparently changed after cellar fermentation. Furthermore, the distances between 7C and others were larger, which can be explained by the winemaker's view that the basic liquors of 4th and 5th rounds were indeed different from that of the 7th round. A similar result was obtained for sample 6C, albeit less dramatic. The structure of the volatile compounds of 6C and 7C had obvious difference to former ones.

As shown in Figure 3b, all the C samples almost gathered to one point in terms of bacteria, indicating that the structure of bacteria became similar to each other after fermentation. If DA45 is the distance of 4A to 5A, DA56 indicated the distance of 5A to 6A, and so on. As shown in Figure 3b, DA45 and DB45 were small and the relationships of the distances were as follows: DA56 > DA67 > DA45; DB56 > DB67 > DB45, which indicated that the A samples of 4th and 5th rounds were similar as well as B samples, that the gaps were larger between 5th and 6th rounds, and that smaller gaps existed between the 6th and 7th rounds. The same trends of A and B samples increased the credibility of these observations. These results indicated that the bacterial diversity could not be adjusted back after the 5th round. Bacteria of A and B samples changed considerably after the 5th round, while the volatile compounds were influenced negligibly at that moments. This was because cooling and stacking did not last long, but cellar fermenting continued for 1 month, which resulted in the large difference in volatile compound composition. As the volatile compounds were generated by the microorganisms, this might explain the result shown in Figure 3a. These results were also in agreement with the empirical viewpoint.

The stacked samples of the 6th round were different from those of the 4th and 5th rounds, and hence, the base liquor distilled from the fermented grains after 1 month had unequal flavor, the liquors of 4th and 5th rounds became “Da Hui” and latter ones became “Xiao Hui.” Up to the 7th round, as the gap increased and the flavor worsened, the liquor produced was the so‐called “Zhui Zao” (Yang et al., 2004). After the 5th round, the quality of the liquor presented a decreasing trend of liquor quality. The quality of base liquors distilled from different rounds presented a single peak model, and the distilled liquors in the middle round were best. This phenomenon warrants further investigation.

### 3.5 Relationship between volatile compounds and dominant strains

**FIGURE 4:**
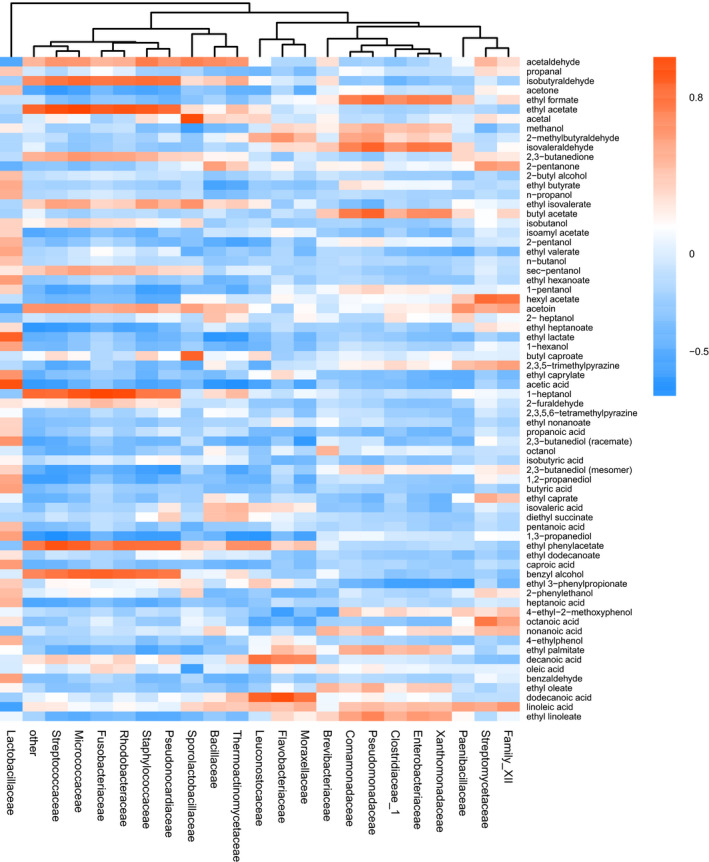
The heat map of volatile compounds and bacterial relative abundance on family level. Longitudinal data represented for volatile compounds and horizontal data represented bacteria at family level. The color of cubes presented the Pearson correlation coefficient between volatile compounds and bacterial abundance

We selected all bacteria with relative abundance >0.1% to analyze the relationship with the volatile compounds (Figure 4). The bacteria were clustered using the Pearson correlation coefficient. The selected 22 families were divided into seven small parts or three large parts using cluster analysis, which were represented by the branches on the top of the heat map.

Many substances are present in several types of Baijiu, the differences in which may explain the differences in flavor. Esters, such as ethyl acetate, ethyl hexanoate, ethyl butyrate, and ethyl lactate, are considered important factors contributing to flavor. Ethyl acetate was positively related to the presence of *Streptococcaceae, Micrococcaceae, Fusobacteriaceae, Rhodobacteraceae, Staphylococcaceae*, and *Pseudonocardiaceae*, which were present in one cluster, indicating that these bacteria possessed similar metabolic pathways and might be the keys for adjusting the yield of ethyl acetate. Ethyl butyrate was negatively related to *Thermoactinomycetaceae* and *Bacillaceae*, which were abundant in the initial stage, and was positively related to *Lactobacillaceae*, which was dominant after cellar fermentation. As ethyl butyrate presented different relationships with these three families, it may be an important flavoring substance mainly generated during fermentation. The trend of ethyl hexanoate was similar to that of ethyl butyrate, which showed positive relationship with *Lactobacillaceae*. Ethyl lactate correlated negatively with most bacteria, but showed strong positive correlation with *Lactobacillaceae,* which was reasonable, as lactic acid is a product of *Lactobacillaceae* and a precursor of ethyl lactate (da Silva Sabo et al., 2014). The above four ethyl esters were the major flavor substance in strong flavor liquor, another major type of Baijiu (Shen, 2003).

**FIGURE 5:**
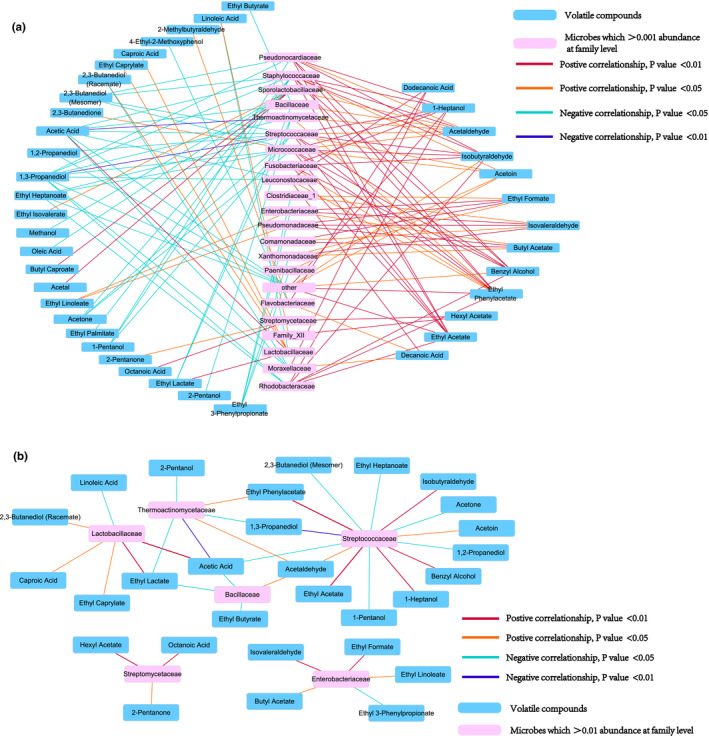
Network shows significant correlations between bacteria and volatile compounds. (a) Bacteria beyond 0.1% abundance. (b) Bacteria beyond 1% abundance

In the cluster, *Lactobacillaceae* was distant from others. It completely dominated the fermentation afterward, indicating that it might play a critical role in CSSL processing. The relationship between volatile compounds and bacterial abundance was analyzed using Pearson correlation analysis, and the bacteria with relative abundance >0.1% are shown using a network diagram (Figure 5a). As shown in Figure 5a, ethyl acetate and ethyl butyrate, two important aromatic compounds, were significantly related to bacteria. Ethyl acetate is an important compound in Moutai (Xiao et al., 2014), a typical CSSL in China. Ethyl acetate was detected in all samples, especially in the 4th round, and showed significant positive correlations with *Fusobacteriaceae, Micrococcaceae, Staphylococcaceae, Streptomycetaceae,* and *Pseudonocardiaceae*. Acetic acid showed significant positive correlation with *Lactobacillaceae* and significant negative correlation with *Thermoactinomycetaceae, Streptococcaceae*, and *Bacillaceae* (Figure 5b). *Lactobacillaceae* and *Bacillaceae* were reported to be the dominant bacteria in *Daqu* of CSSL (Jin et al., 2019). The acids with high boiling point were the chief flavor substances of CSSL (Fan and Xu, 2012). Acetic acid, with high boiling point and a typical strong smell, showed positive relationship with *Lactobacillaceae*. The above results suggested that acetic acid could be one of the most important contributors to CSSL flavor.

In this study, *Streptococcaceae* showed significant correlations with 13 volatile compounds, the average relative abundance of which was 1.5%, and 10 of these were unique associations. Together with *Lactobacillaceae, Streptococcaceae* is also found in milk source fermented food, which imparts a unique flavor to fermented food (Leite et al., 2012; Marino et al., 2019; Motato et al., 2017). For determining its network with multiple volatile compounds and its performance in fermented foods, the metabolic characteristics of *Streptococcaceae,* in addition to its interaction with *Lactobacillaceae* in CSSL, warrant further investigations.

*Enterobacteriaceae* mainly existed in the cooled and stacked stages and its abundance increased with the rounds in this study (Figure 2d). It was a predominant bacterium in CSSL *Daqu* (Jin et al., 2019) and was also found during Chinese rice wine production (Fang et al., 2015). It correlated negatively with ethyl linoleate, ethyl phenylacetate, ethyl formate, ethyl butyrate, and isovaleraldehyde. However, previous studies have mostly focused on the pathogenicity and tolerance of *Enterobacteriaceae* (Osaili et al., 2018). As *Enterobacteriaceae* negatively correlated with many types of ethyl ester compounds, immediate action or indirect action regarding ethyl ester metabolism requires further investigations. Furthermore, isovaleraldehyde has a malty, fruity, and cocoa‐like odor and is often used as flavor additive in food (Tian et al., 2007). Therefore, *Enterobacteriaceae* may affect CSSL flavor via the production of isovaleraldehyde.

Bacteria with abundance <0.1% (other) showed significant positive correlations with ethyl phenylacetate, ethyl acetate, and benzyl alcohol, and significant negative correlations with ethyl heptanoate, 1,3‐propanediol, 1,2‐propanediol, and acetic acid. Acetic acid can be converted to ethyl acetate via esterification (Gurav and Bokade, 2010), which may explain their inverse relationships with low‐abundance bacteria.

2,3,5,6‐Tetramethylpyrazine and 2,3,5‐trimethylpyrazine are considered the main flavoring substance (Sun et al., 2015). In this study, 2,3,5,6‐tetramethylpyrazine was almost negatively related to all bacteria, albeit not significant. This might be because the abundance of the bacterium that produces tetramethylpyrazine was extremely low, or some abundant bacterium may produce it only in the narrow stage. As the time of sampling in this study was discontinuous, some information might have been lost. 2,3,5‐Trimethylpyrazine correlated positively with *Streptomycetaceae, Paenibacillaceae,* and *Family_XII* (o:*Bacillales*), albeit without statistical significance.

### 3.6 Canonical correlation analysis of bacteria and environmental factors

The relationship between environmental factors and bacterial abundance was analyzed using canonical correlation analysis (CCA). CCA is a classical way of describing the relationship between environmental factors and microorganisms. In the fermentation industry, biochemical factors such as pH are significant parameters that can be adjusted to meet production needs (Peng et al., 2016).

**FIGURE 6:**
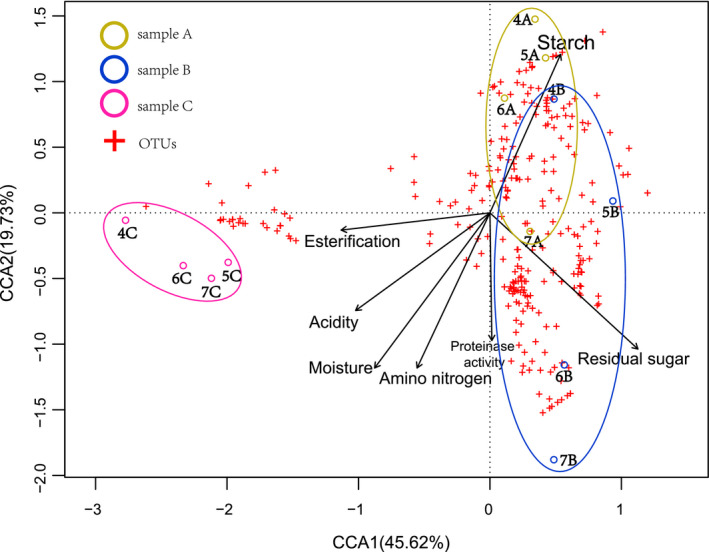
The CCA analysis of bacterial diversity and biochemistry compounds. Totally 12 samples, 7 environmental factors and 267 OTUs involved

The diagram of CCA expresses the information shown in Figure 6. Cross icons indicate OTUs, which added up to 267. The biochemical factors are represented by black arrows, the length and angle of which indicate the size and direction of correlation. The ellipse indicated the fluctuation range of one stage of different rounds.

The OTUs can be approximately divided into two parts. The major cluster on the right side of Figure 6 contained 239 OTUs, while the small cluster contained 28 OTUs, of which 25 OTUs belonged to *Lactobacillaceae*. As mentioned before, the angle of the cross star and arrow determined the Pearson correlation coefficient, and the acute angle represented a positive relationship and vice versa. These indicated that *Lactobacillaceae* showed significant negative correlation with the starch and residual sugar and positive correlation with esterification and acidity. After cellar fermentation, acids in each round have a same trend of increasing (Table S1). Combined with *Lactobacillaceae* becoming the absolute dominant species in cellar fermentation stage, we speculate that there are two aspects, one is that *Lactobacillaceae* can produce acid substances, these products accumulated in the system. On the other hand, with the pH of the fermentation system further decreased, acid‐resistant microorganisms gradually replaced those were not. In addition, *Lactobacillaceae* was close to the four samples at the fermented stage of all rounds. Other OTUs mainly existed in the cooled and stacked stages.

The sizes of the ellipses illustrated that stacking could improve the diversity of bacteria, while cellar fermentation made it homogeneous. The result was identical to that reported previously (Cheng et al., 2014). Stacking was maintained for about 3 days to enrich the microorganisms for cellar fermentation, leading to diversification of bacterial diversity. Thus, the traditional technology was used in multiple rounds of fermentation to prevent some bacteria from occupying the main part. The presence of volatile compounds generated via the varied metabolic pathways in microorganisms was the basis of unique aroma (Xu et al., 2010).

To summarize, a dynamic contour of CSSL was drawn based on the obtained results from this work, aiming to investigate the empirical points of CSSL manufacture and visualize the relationship between bacteria and volatile compounds besides environmental factors.

## 4 CONCLUSION

Bacterial community structure, volatile compound content, and environmental factors of CSSL fermented grains in different stages during the 4th to 7th rounds were assessed in this study. *Bacillaceae, Thermoactinomycetaceae*, and *Enterobacteriaceae* mainly existed before cellar fermentation and were subsequently replaced by *Lactobacillaceae*. PCA of volatile compounds supported the popular belief that liquor quality changes with increase in fermentation rounds. PCoA of bacteria explained the phenomenon from the perspective of microbial metabolism. The Pearson correlation coefficient provided a preliminary profile between bacterial relative abundance and volatile compounds then revalidate the former viewpoint. CCA of environmental factors and bacteria provided potential methods for adjusting CSSL manufacture and indicated that *Lactobacillaceae* was an extremely crucial microbe in this process. This study provided improved understanding regarding the dynamic changes in the bacterial community and volatile compounds of CSSL. Interactions between key microorganisms, more detailed metabolic profiles and combination analysis of sensory and numeric value might be the main research directions of CSSL.

## CONFLICT OF INTEREST

The authors declare that they do not have any conflict of interest.

## ETHICAL APPROVAL

This study does not involve any human or animal testing.

## INFORMED CONSENT

Written informed consent was obtained from all study participants.

## Supporting information

Fig S1

Tab S1

Tab S2

Tab S3
