# Supplementary figures and images for: Dynamic changes of volatile compounds and bacterial diversity during fourth to seventh rounds of Chinese soy sauce aroma liquor
Source: Food Sci Nutr. 2021 May 12;9(7):3500–11. doi: 10.1002/fsn3.2291 (PMC8269578; doi:10.1002/fsn3.2291)

**a**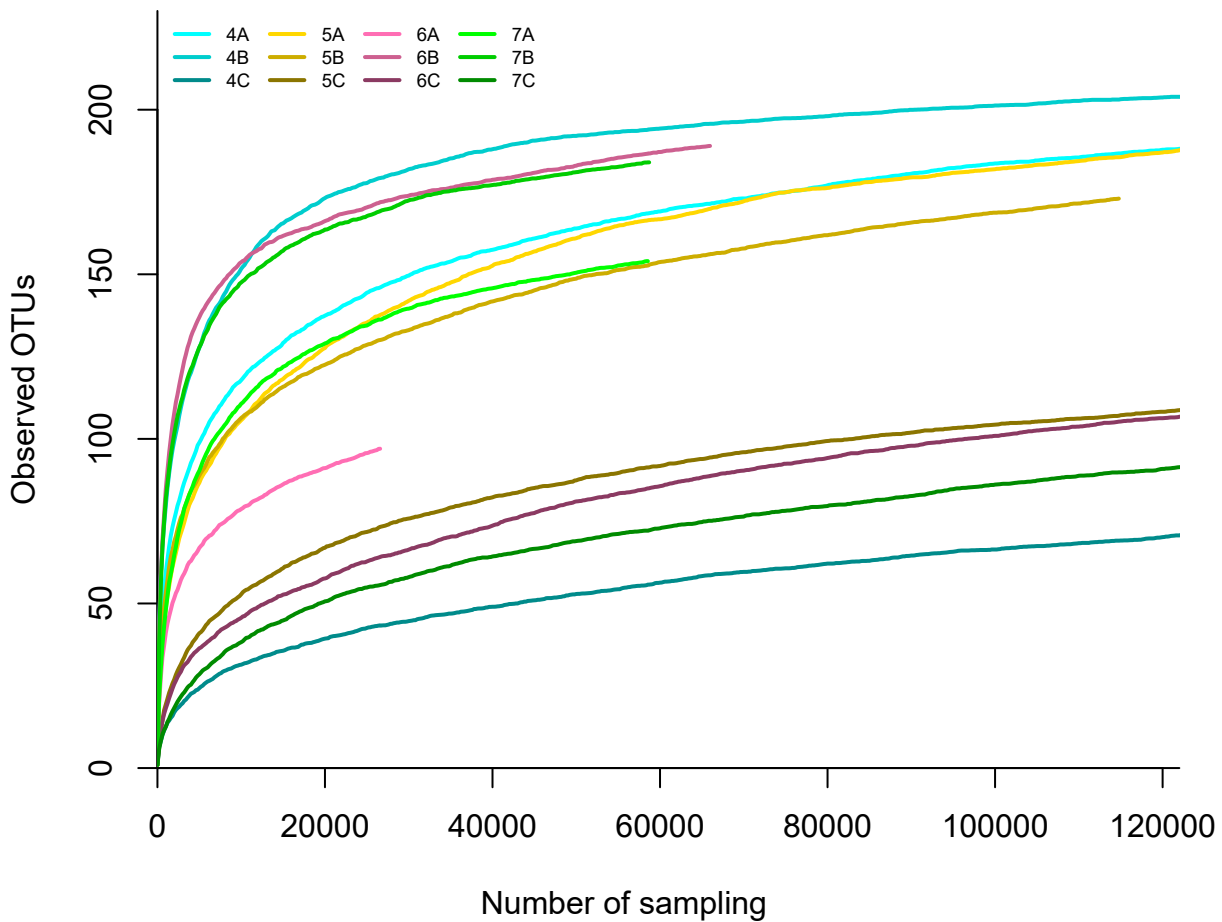**b**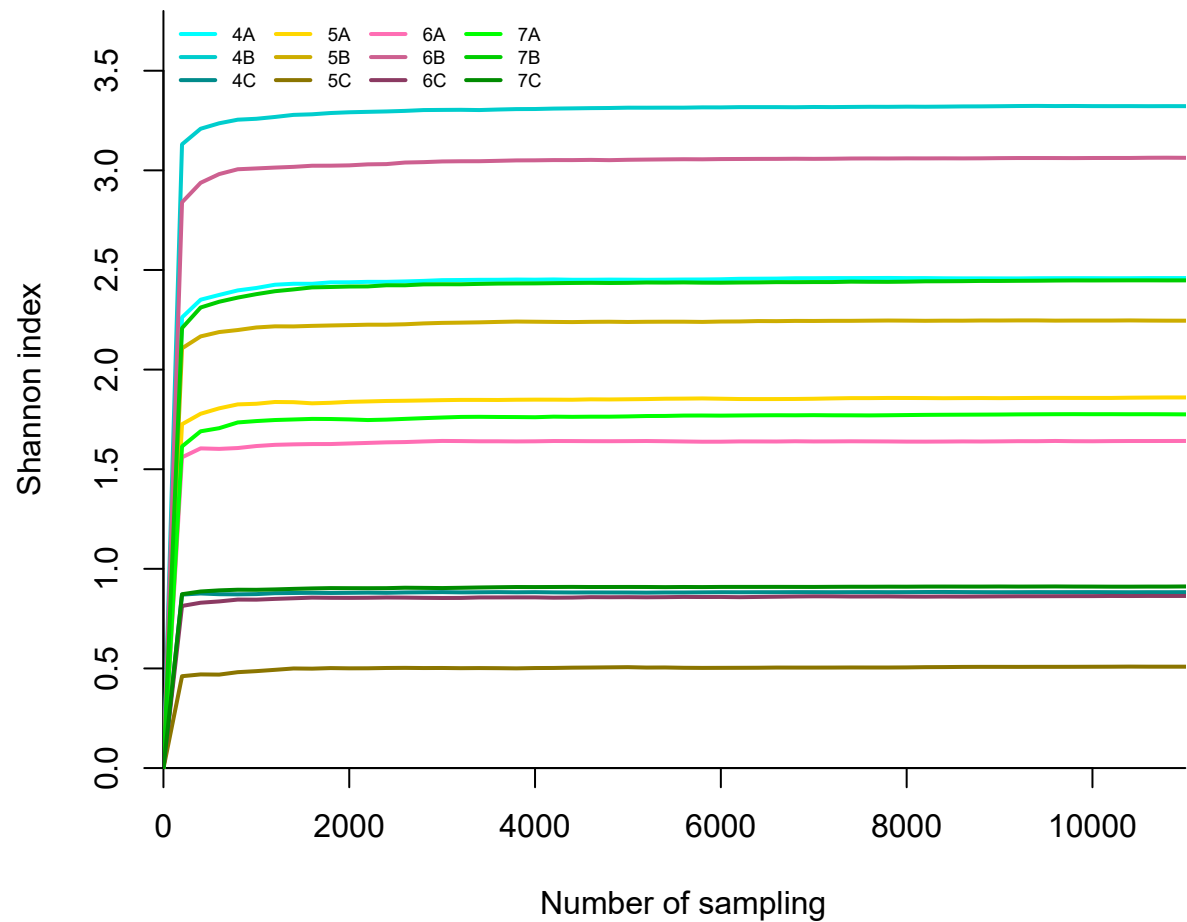

Supplement: Supplementary file 1 — Fig S1 [file FSN3-9-3500-s001.pdf]
