# Supplementary material for: Dynamic changes of volatile compounds and bacterial diversity during fourth to seventh rounds of Chinese soy sauce aroma liquor
Source: Food Sci Nutr. 2021 May 12;9(7):3500–11. doi: 10.1002/fsn3.2291 (PMC8269578; doi:10.1002/fsn3.2291)
Supplement: Supplementary file 2 — Tab S1 [file FSN3-9-3500-s003.docx]

Table S1 Volatile compounds identified in CSSL 4^th^ to 7^th^ rounds fermented grains

| Volatile compounds | 4A | 4B | 4C | 5A | 5B | 5C | 6A | 6B | 6C | 7A | 7B | 7C |
| --- | --- | --- | --- | --- | --- | --- | --- | --- | --- | --- | --- | --- |
| Ethyl formate^a^ | 0.72 | 0.26 | 1.5 | 1.68 | 2.43 | 1.06 | 1.93 | 2.95 | 3.22 | 5.02 | 7.45 | 4.64 |
| Ethyl acetate | 36.26 | 20.13 | 4.35 | 2.4 | 1.34 | 3.65 | 5.19 | 1.29 | 2.64 | 1.46 | 1.08 | 3.6 |
| Ethyl butyrate | 0.13 | 0.2 | 0.41 | 0.2 | 0.13 | 0.11 | 0.08 | 0.06 | 0.17 | 0.12 | 0.35 | 0.6 |
| Ethyl linoleate | ND | 0.04 | 1.82 | 0.92 | 1.47 | 4.25 | 3.38 | 2.64 | 3.84 | 5.73 | 6.26 | 2.19 |
| Ethyl oleate | 0.03 | 0.03 | 0.77 | 0.21 | 0.45 | 1.79 | 1.71 | 1.08 | 10.81 | 10.83 | 7.74 | 4.19 |
| Ethyl isovalerate | 0.18 | 0.28 | 0.06 | 0.13 | 0.11 | ND | 0.06 | 0.18 | 0.11 | ND | ND | 0.09 |
| Ethyl palmitate | 0.2 | 0.04 | 1.05 | 1.01 | 0.79 | 3.05 | 2.78 | 1.46 | 2.28 | 2.45 | 3.38 | 1.4 |
| Diethyl succinate | 0.11 | 0.09 | 0.63 | 3.44 | 0.23 | 0.11 | 0.82 | ND | ND | ND | ND | 0.3 |
| Ethyl phenylacetate | 21.54 | 17.49 | ND | ND | ND | 0.07 | 14.6 | 1.17 | ND | ND | ND | ND |
| Ethyl dodecanoate | ND | 0.03 | 0.03 | 0.05 | ND | ND | ND | ND | ND | ND | ND | 0.04 |
| Ethyl 3-phenylpropionate | 0.09 | 0.16 | 0.14 | 0.09 | 0.07 | 0.11 | 0.16 | ND | 0.18 | 0.03 | ND | 0.03 |
| Ethyl hexanoate | 0.29 | 0.16 | 0.71 | 0.15 | 0.41 | 0.45 | 0.37 | 0.08 | 0.24 | 0.29 | 0.21 | 1.86 |
| Ethyl caprate | 1.12 | 0.74 | 1.73 | 1.73 | 1.93 | 1.54 | 1.33 | 1.66 | 1.05 | 1.06 | 0.9 | 1.24 |
| Butyl acetate | 0.06 | ND | 0.07 | 0.12 | 0.15 | 0.07 | ND | ND | 0.11 | 0.28 | 0.38 | 0.09 |
| Ethyl heptanoate | 0.12 | 0.19 | 0.72 | 0.61 | 0.55 | 0.48 | 0.36 | 0.44 | 0.21 | 0.32 | 0.25 | 0.47 |
| Ethyl lactate | 1.66 | 1.1 | 73.06 | 6.79 | 3.56 | 29.59 | 3.41 | 1.67 | 22.63 | 4.54 | 2.66 | 53.57 |
| Isoamyl acetate | ND | ND | 0.25 | 0.15 | 0.09 | 0.16 | 0.15 | 0.13 | ND | 0.04 | ND | 0.11 |
| Butyl caproate | ND | 0.27 | 0.04 | 0.09 | 0.05 | 0.06 | ND | ND | ND | ND | 0.06 | ND |
| Hexyl acetate | 0.04 | 0.17 | 0.09 | 0.06 | 1.07 | 0.47 | 0.48 | 0.36 | 0.31 | 0.41 | 0.44 | 0.47 |
| Ethyl nonanoate | ND | 0.03 | 0.08 | 0.12 | 0.07 | ND | 0.22 | 0.05 | 0.18 | 0.25 | 0.08 | 0.63 |
| Ethyl caprylate | 0.03 | 0.06 | 0.18 | 0.09 | 0.04 | 0.11 | 0.28 | 0.08 | 0.24 | 0.04 | ND | 0.39 |
| Ethyl valerate | 0.19 | ND | 0.34 | 0.23 | 0.07 | 0.15 | 0.1 | ND | 0.09 | 0.1 | 0.08 | 0.35 |
| Esters | 62.77 | 41.47 | 88.03 | 20.27 | 15.01 | 47.28 | 37.41 | 15.3 | 48.31 | 32.97 | 31.32 | 76.26 |
|  |  |  |  |  |  |  |  |  |  |  |  |  |
| Methanol | 1.26 | 0.2 | 0.77 | 0.58 | 0.24 | 2.67 | 2.21 | 1.8 | 2.23 | 2.59 | 2.92 | 2.29 |
| 2-butyl alcohol | 0.43 | 0.38 | 0.15 | 0.32 | 0.2 | 0.45 | 0.41 | ND | 0.27 | 0.36 | 0.19 | 2.25 |
| N-propanol | 3.6 | 3.85 | 2.12 | 0.88 | 1.17 | 1.33 | 0.67 | 0.8 | 5.71 | 0.92 | 0.93 | 35.55 |
| Isobutanol | 1.13 | 1.18 | 0.8 | 0.19 | 0.92 | 0.6 | 0.45 | 0.45 | 0.38 | 0.33 | 0.44 | 1.76 |
| 2-pentanol | 0.23 | 0.12 | 0.27 | 0.19 | 0.29 | 0.2 | 0.15 | 0.19 | 0.7 | 0.43 | 0.43 | 0.64 |
| N-butanol | 0.15 | 0.08 | 0.96 | 0.1 | 0.14 | 0.23 | 0.06 | 0.09 | ND | 0.04 | 0.05 | ND |
| Isoamyl alcohol | 4.68 | 3.96 | 2.47 | 0.56 | 2.36 | 1.38 | 1.61 | 0.83 | 1.02 | 0.86 | 1.23 | 5.44 |
| 1-pentanol | 1.6 | 1.53 | 1.89 | 1.8 | 2.21 | 2.19 | 2.17 | 2.11 | 2.52 | 2.61 | 2.61 | 3.28 |
| 2- heptanol | 0.33 | 0.11 | 0.31 | 2.6 | 0.3 | 0.12 | 0.13 | 3.52 | 0.5 | 0.9 | 0.07 | 0.1 |
| 1-hexanol | 0.09 | 0.05 | 0.32 | 0.13 | 0.2 | 0.14 | 0.07 | 0.1 | 0.08 | 0.12 | 0.13 | 0.5 |
| 1-heptanol | 0.78 | 0.03 | ND | ND | 0.15 | 0.03 | 0.05 | 0.12 | ND | ND | ND | ND |
| Octanol | 0.05 | 0.02 | 0.38 | 0.34 | 0.14 | 0.14 | 0.12 | 0.24 | 0.09 | 0.42 | 0.15 | 0.16 |
| 2,3-butanediol (racemate) | 9.08 | 9.67 | 14.79 | 11.08 | 12.78 | 11.16 | 9.06 | 6.81 | 12.16 | 12.09 | 10.62 | 15.44 |
| 2,3-butanediol (mesomer) | 6.19 | 8.21 | 10.68 | 9.36 | 15.22 | 10.5 | 9.52 | 9.99 | 16.37 | 15.77 | 16.83 | 20.87 |
| 1,2-propanediol | 27.21 | 23.39 | 49.59 | 40.03 | 42.68 | 36.76 | 31.76 | 25.26 | 35.54 | 37.45 | 34.92 | 52.97 |
| 1,3-propanediol | 0.18 | 0.05 | 37.02 | 29.45 | 31.1 | 23.45 | ND | 15.61 | 36.09 | 30.23 | 27.67 | 55.65 |
| Benzyl alcohol | 0.36 | 0.14 | 0.12 | 0.12 | 0.04 | 0.05 | 0.03 | ND | 0.04 | 0.08 | 0.05 | 0.17 |
| 2-phenylethanol | 0.87 | 0.99 | 1.00 | 0.46 | 1.05 | 0.66 | 0.52 | 0.54 | 0.74 | 0.52 | 0.76 | 1.21 |
| Alcohols | 58.22 | 53.96 | 123.64 | 98.19 | 111.19 | 92.06 | 58.99 | 68.46 | 114.44 | 105.72 | 100 | 198.28 |
|  |  |  |  |  |  |  |  |  |  |  |  |  |
| Acetic acid | 21.61 | 23.02 | 112.66 | 56.66 | 39.18 | 89.81 | 25.56 | 40.08 | 117 | 47.62 | 22.02 | 104.78 |
| Propanoic acid | 2.27 | 1.71 | 5.37 | 6.08 | 2.64 | 1.64 | 1.19 | 2.86 | 3.44 | 1.52 | 1.12 | 3.72 |
| Isobutyric acid | 0.57 | 0.57 | 0.97 | 1.03 | 0.48 | 0.33 | 0.29 | 0.64 | 0.6 | 0.44 | 0.32 | 0.54 |
| Butyric acid | 0.74 | 0.72 | 2.76 | 2.6 | 0.73 | 0.78 | 0.5 | 1.12 | 7.4 | 1.12 | 0.42 | 2.02 |
| Isovaleric acid | 0.77 | 0.74 | 1.43 | 2.77 | 1.24 | 0.71 | 2.06 | 1.24 | 1.3 | 0.54 | 0.54 | 0.94 |
| Pentanoic acid | 0.49 | 0.04 | 0.72 | 0.78 | 0.49 | 0.21 | 0.73 | 0.65 | 1.9 | 0.54 | 0.44 | 1.04 |
| Caproic acid | 0.96 | 0.37 | 3.51 | 3.21 | 1.72 | 1.42 | 0.81 | 2.23 | 9.84 | 1.76 | 1.34 | 3.8 |
| Heptanoic acid | 0.04 | 0.1 | 0.19 | 0.36 | 0.29 | 0.26 | 0.26 | 0.33 | 0.94 | 0.24 | 0.22 | 0.44 |
| Octanoic acid | 1.08 | 0.24 | 2.13 | 0.67 | 3.29 | 0.56 | ND | 0.56 | 1.44 | 0.56 | 0.4 | 1.43 |
| Nonanoic acid | 0.1 | ND | 0.21 | 0.21 | 0.32 | 0.07 | ND | ND | ND | 0.36 | 0.28 | ND |
| Decanoic acid | 0.56 | 0.51 | 0.66 | 0.18 | 0.15 | 0.33 | 1.03 | 0.32 | 0.22 | 0.24 | 0.4 | 0.38 |
| Oleic acid | 4.52 | 0.17 | 1.81 | 2.46 | 3.27 | 2.49 | 3.47 | 2.88 | 3.9 | 4.56 | 3.58 | 4.99 |
| Dodecanoic acid | 0.53 | 0.91 | 0.37 | 0.31 | 0.59 | 0.56 | 2.56 | 0.46 | 0.64 | 0.96 | 0.98 | 0.84 |
| Linoleic acid | 12.79 | 13.73 | 9.61 | 7.52 | 18.94 | 3.82 | 17.05 | 12.66 | 10.36 | 13.58 | 17.72 | 11.83 |
| Acids | 47.03 | 42.83 | 142.4 | 84.84 | 73.33 | 102.99 | 55.51 | 66.03 | 158.98 | 74.04 | 49.78 | 136.75 |
|  |  |  |  |  |  |  |  |  |  |  |  |  |
| Acetaldehyde | 4.69 | 4.88 | 0.76 | 3.16 | 4.81 | 1.51 | 1.54 | 1.63 | 1.07 | 2.91 | 1.22 | 1.51 |
| Propanal | 0.21 | 0.07 | 0.19 | 0.08 | 0.28 | 0.43 | 0.1 | 0.06 | 0.14 | 0.15 | 0.21 | 0.18 |
| Isobutyraldehyde | 0.29 | 0.16 | 0.07 | 0.09 | 0.09 | 0.15 | 0.08 | ND | ND | 0.11 | ND | ND |
| Acetal | ND | 6.3 | 0.14 | 1.94 | 3.04 | 0.87 | 1.15 | 0.53 | ND | 1.66 | 0.66 | 1.37 |
| 2-methylbutyraldehyde | 0.09 | 0.09 | 0.16 | 0.33 | 0.16 | 0.29 | 0.56 | ND | 0.11 | 0.25 | 0.52 | 0.26 |
| Isovaleraldehyde | 0.12 | ND | 0.17 | 0.09 | 0.31 | 0.58 | 0.9 | 0.72 | 1.15 | 1.89 | 2.4 | 0.46 |
| Benzaldehyde | 0.71 | 0.32 | 2.91 | ND | 2.85 | 2.21 | 0.83 | 0.84 | 1.24 | 0.97 | 1 | 10.29 |
| 2-furaldehyde | 1.05 | 0.37 | 0.45 | 0.56 | 0.63 | 0.33 | 0.27 | 0.16 | 0.38 | 0.37 | 0.34 | 1.48 |
| Aldehydes | 7.16 | 12.19 | 4.85 | 6.25 | 12.17 | 6.37 | 5.43 | 3.94 | 4.09 | 8.31 | 6.35 | 15.55 |
| Acetone | ND | ND | 0.2 | 0.23 | 0.27 | 0.42 | 0.08 | ND | 0.15 | 0.23 | 0.23 | 0.28 |
| 2,3-butanedione | 0.35 | 0.22 | ND | ND | 0.21 | ND | ND | 0.24 | ND | ND | ND | 0.29 |
| 2-pentanone | ND | ND | 0.03 | 0.1 | 0.12 | ND | 0.05 | ND | ND | 0.07 | 0.06 | 0.03 |
| Acetoin | 5.4 | 4.79 | 0.76 | 1.07 | 5.17 | 0.33 | 1.11 | 3.94 | 0.18 | 0.9 | 2.85 | 1.34 |
| Ketones | 5.75 | 5.01 | 0.99 | 1.4 | 5.77 | 0.75 | 1.24 | 4.18 | 0.33 | 1.2 | 3.14 | 1.94 |
|  |  |  |  |  |  |  |  |  |  |  |  |  |
| 4-ethyl-2-methoxyphenol | 0.21 | 0.26 | 0.26 | 0.56 | 0.41 | 0.24 | 0.04 | 0.28 | 0.37 | ND | 0.49 | 0.33 |
| 4-ethylphenol | 0.04 | 0.1 | 1.14 | 1.03 | 0.41 | 56.96 | 52.25 | 0.91 | 1.48 | 1.32 | 1.58 | 104.51 |
| Phenols | 0.25 | 0.36 | 1.4 | 1.59 | 0.82 | 57.2 | 52.29 | 1.19 | 1.85 | 1.32 | 2.07 | 104.84 |
|  |  |  |  |  |  |  |  |  |  |  |  |  |
| 2,3,5-trimethylpyrazine | 0.27 | ND | 0.38 | 0.43 | 0.52 | 0.31 | 0.26 | 0.46 | 0.3 | 0.4 | 0.41 | 0.33 |
| 2,3,5,6-tetramethylpyrazine | 0.04 | ND | 0.05 | 0.24 | 0.07 | 0.05 | 0.05 | 0 | 0.04 | 0.07 | 0.05 | 0.21 |
| Pyrazines | 0.31 | ND | 0.43 | 0.67 | 0.59 | 0.36 | 0.31 | 0.46 | 0.34 | 0.47 | 0.46 | 0.54 |

**mg/100mL**

ND：not detected
