# Supplementary material for: Dynamic changes of volatile compounds and bacterial diversity during fourth to seventh rounds of Chinese soy sauce aroma liquor
Source: Food Sci Nutr. 2021 May 12;9(7):3500–11. doi: 10.1002/fsn3.2291 (PMC8269578; doi:10.1002/fsn3.2291)
Supplement: Supplementary file 3 — Tab S2 [file FSN3-9-3500-s004.docx]

| Sample_ID | PE_Reads | Raw_Tags | Clean_Tags | AvgLen(bp) | GC(%) | Q20(%) | Q30(%) | Effective(%) |
| --- | --- | --- | --- | --- | --- | --- | --- | --- |
| 4A | 212621 | 201025 | 169161 | 466 | 55.08 | 96.93 | 94.18 | 79.56 |
| 4B | 226871 | 215098 | 189592 | 468 | 54.21 | 96.98 | 94.32 | 83.57 |
| 4C | 180351 | 173255 | 157136 | 469 | 50.28 | 96.91 | 94.25 | 87.13 |
| 5A | 174177 | 166393 | 140475 | 469 | 55.43 | 97.02 | 94.34 | 80.65 |
| 5B | 186475 | 168674 | 129094 | 467 | 56.65 | 96.93 | 94.17 | 69.23 |
| 5C | 226497 | 206292 | 179970 | 469 | 50.13 | 95.12 | 91.08 | 79.46 |
| 6A | 37846 | 34387 | 29836 | 469 | 55.31 | 95.18 | 91.02 | 78.84 |
| 6B | 104034 | 92809 | 79918 | 468 | 54.91 | 94.97 | 90.65 | 76.82 |
| 6C | 193927 | 177547 | 151739 | 469 | 50.22 | 95.19 | 91.19 | 78.25 |
| 7A | 80957 | 73754 | 64321 | 469 | 54.86 | 94.92 | 90.57 | 79.45 |
| 7B | 85558 | 77860 | 68516 | 468 | 54.39 | 95.09 | 90.96 | 80.08 |
| 7C | 194710 | 179257 | 158218 | 469 | 50.41 | 95.21 | 91.21 | 81.26 |

Table S2 The quality and statistics of sequencing

Q30: Error probability less than 0.1%

Q20: Error probability less than 1%.

Effective: Clean Tags/PE Reads *100%
