# Supplementary material for: Dynamic changes of volatile compounds and bacterial diversity during fourth to seventh rounds of Chinese soy sauce aroma liquor
Source: Food Sci Nutr. 2021 May 12;9(7):3500–11. doi: 10.1002/fsn3.2291 (PMC8269578; doi:10.1002/fsn3.2291)
Supplement: Supplementary file 4 — Tab S3 [file FSN3-9-3500-s002.docx]

| Samples | Observed OTUs | Ace | Chao1 | Shannon | Simpson | Goods  coverage |
| --- | --- | --- | --- | --- | --- | --- |
| 4A | 192 | 206.037 | 203.053 | 2.467 | 0.812 | 0.999859 |
| 4B | 208 | 216.040 | 221.000 | 3.328 | 0.931 | 0.999924 |
| 4C | 75 | 100.130 | 103.111 | 0.887 | 0.520 | 0.999844 |
| 5A | 189 | 204.312 | 202.143 | 1.869 | 0.691 | 0.999812 |
| 5B | 173 | 197.164 | 196.625 | 2.252 | 0.812 | 0.999756 |
| 5C | 115 | 127.115 | 124.500 | 0.510 | 0.181 | 0.999887 |
| 6A | 97 | 119.414 | 118.000 | 1.644 | 0.650 | 0.999210 |
| 6B | 189 | 210.133 | 210.000 | 3.064 | 0.894 | 0.999682 |
| 6C | 111 | 137.701 | 140.000 | 0.866 | 0.438 | 0.999794 |
| 7A | 154 | 169.931 | 182.111 | 1.780 | 0.664 | 0.999607 |
| 7B | 184 | 192.346 | 192.750 | 2.457 | 0.713 | 0.999744 |
| 7C | 97 | 131.401 | 122.200 | 0.913 | 0.520 | 0.999811 |

Table S3 The bacterial alpha diversity of 4^th^ to 7^th^ CSSL fermented grains
